# Harnessing the Single-Session Intervention approach to promote scalable implementation of evidence-based practices in healthcare

**DOI:** 10.3389/frhs.2022.997406

**Published:** 2022-09-23

**Authors:** Jessica L. Schleider, Rinad S. Beidas

**Affiliations:** ^1^Department of Psychology, Stony Brook University, Stony Brook, NY, United States; ^2^Department of Medical Ethics and Health Policy, Perelman School of Medicine, University of Pennsylvania, Philadelphia, PA, United States; ^3^Department of Psychiatry, Perelman School of Medicine, University of Pennsylvania, Philadelphia, PA, United States; ^4^Penn Implementation Science Center at the Leonard Davis Institute of Health Economics (PISCE@LDI), University of Pennsylvania, Philadelphia, PA, United States; ^5^Penn Medicine Nudge Unit, University of Pennsylvania Health System, Philadelphia, PA, United States; ^6^Center for Health Incentives and Behavioral Economics, Perelman School of Medicine, University of Pennsylvania, Philadelphia, PA, United States; ^7^Department of Medical Social Sciences, Feinberg School of Medicine, Northwestern University, Evanston, IL, United States

**Keywords:** implementation science, implementation strategy, Single-Session Intervention, Theoretical Domains Framework, behavior change

## Abstract

Effective implementation of evidence-based practices often involves multi-level strategies targeting individual-, organizational-, and system-level determinants of change. Although these multi-level implementation approaches can successfully facilitate EBP uptake, they tend to be complex and resource intensive. Accordingly, there is a need for theory-driven, generalizable approaches that can enhance efficiency, cost-effectiveness, and scalability of existing implementation approaches. We propose the Single-Session Intervention approach as an unexplored path to developing low-cost and scalable implementation strategies, especially those targeting individual-level behavior change. We argue that single-session strategies (S3) for implementation, which can simultaneously target myriad barriers to individual behavior change, may promote clinicians' EBP uptake and sustainment in a manner that is low-resource and scalable. We first overview the evidence-base supporting the Single-Session Intervention approach for patient-level outcomes; situate this approach within the implementation science literature by outlining its intersections with a leading framework, the Theoretical Domains Framework (TDF), as an exemplar; and illustrate how the TDF might directly inform the design and evaluation of single-session strategies for EBP implementation. Overall, single-session strategies (S3) for implementation reflect a promising but yet-to-be-tested means of streamlining and scaling individual-level behavior change efforts in healthcare settings. Future partnered research is needed to gauge the potential of this approach across diverse clinical and community contexts.

## Background

Per the widely-touted 17-year gap between the identification and application of evidence-based clinical practices, the so-called research-practice gap reflects a canonically “wicked problem” in healthcare (1, 2). This care gap undermines access to effective treatment across health service sectors, including all levels of care (e.g., acute, ambulatory) and across disease areas (e.g., psychiatry, oncology, primary care). In response to this challenge, implementation science has emerged as a discipline focused on systematically studying methods to increase the adoption, use, and sustainment of evidence-based practices (EBPs) in settings where care is delivered. Implementation approaches often deploy multi-level strategies targeting individual, organizational, system, and sociopolitical determinants (i.e., barriers and facilitators) to individual behavior change (3). In many cases, these multi-level and multi-faceted implementation approaches have facilitated increases in use of evidence-based clinical care (4). However, they are often costly and complex to sustain—and past implementation science efforts have struggled to support individual, clinician-level behavior change absent expensive and often-infeasible implementation plans (5). When they have been deployed, they are often not theoretically derived, minimizing their potential impact (6) and preventing identification of change mechanisms (7), which has been highlighted as key to strengthening implementation strategies across levels. These gaps highlight the need for approaches that improve the efficiency, cost-effectiveness, capacity for mechanism-identification, and scalability of effective implementation strategies that shape clinician-level change. Ideally, such approaches could easily integrate with implementation strategies at other levels, across diverse settings and contexts. To enhance their broad usability, such approaches should also be generalizable, offering theory-driven guidelines for scaling implementation strategies for widely-varying practice goals.

We argue that the Single-Session Intervention approach (8)—typically applied to increasing the scalability of patient-level clinical interventions—presents an untapped opportunity to improve the scalability of implementation strategies targeting individual clinician behaviors. We propose that single-session strategies (S3) for implementation may efficiently support clinicians' adoption, implementation, and sustainment of EBPs. Although some brief implementation strategies have been developed and examined previously (e.g., a “pre-implementation enhancement strategy” to strengthen the utility of school-based consultation (9); a brief program leveraging parent opinion leaders to support caregivers to pursue evidence-based mental health care for their children (10), prior efforts have not prioritized the scalability and generalizability of brief, targeted implementation strategies. Below, we overview evidence supporting the Single-Session Intervention approach; highlight its natural intersections with a widely-applied implementation science framework, the Theoretical Domains Framework (11); and outline how the development of mechanism-targeted single-session strategies, built for and with specific populations of clinicians and optimized for scalability, may streamline the development and deployment of flexible, low-cost, and targeted implementation strategies that work.

## The Single-Session Intervention approach

Single-Session Interventions (SSIs) are “structured programs that intentionally involve just one visit or encounter with a clinic, provider, or program” (8). To date, they have focused on patient-level clinical interventions and associated outcomes. Often, they target core mechanisms of longer-term healthcare interventions, such as a program teaching a single evidence-based treatment strategy for depression (cognitive reappraisal; behavioral activation). However, their brevity and flexibility augments their immediate, cost-effective scalability. SSIs may be offered as stand-alone interventions or adjunctive supports within broader healthcare systems; they may be delivered by trained providers or *via* digital, self-guided programs; and *via* diverse settings, from classrooms to clinics to smartphones.

SSIs have improved individual-level outcomes spanning many disciplines, including education, medicine, and public health. SSIs have increased motivation to empathize, empathic accuracy, and the number of friendships in college students (12); decreased alcohol consumption among individuals with alcohol use disorder (13); mitigated rates of HIV infection among high-risk adolescents (14); increased distress tolerance and endorsement of positive parenting practices among high-anxiety parents of young children (15); decreased self-hatred and increased intentions to stop self-harming in youth with histories of non-suicidal self-injury (16, 17); produced clinically significant improvements in pain catastrophizing, pain intensity, and pain interference in adults with chronic lower back pain, with non-inferior effects to 8-session cognitive behavioral therapy (18); and significantly reduced 3-month depression, anxiety, hopelessness, and restrictive eating behaviors in a nationwide sample of adolescents (*N* = 2,452) during the COVID-19 pandemic, vs. a supportive-therapy control (19). In a meta-analysis of 50 randomized trials, SSIs significantly reduced youth mental health problems, relative to their respective controls, with effect sizes only slightly smaller than those observed for longer-term and more expensive youth mental health treatments (20, 21).

How do SSIs work? Broadly, they target theory-driven principles and proximal factors that underlie general behavior change—regardless of the distal outcome of interest. For example, Schleider et al. (8) describe a four-component process to designing SSIs capable of spurring behavior change, grounded in basic research in social psychology, education, and marketing. These design features involve (1) including scientific evidence and social-norming data to normalize the users' experiences and boost message credibility; (2) empowering users as “experts;” (3) allowing users to share back what they learn during the intervention, to help others in their community navigate similar challenges; and (4) including narratives from others facing similar challenges. Many SSIs also guide users to develop an “action plan” for using the new skill, to strengthen motivation and self-efficacy in future strategy use (19, 22, 23). These design principles reflect insights from participatory action research, which highlights the benefits of empowering individuals to “expert” positions (24), which is consistent with implementation science approaches; self-determination theory, which suggests that boosting feelings of competence, agency, and relatedness can motivate adaptive behavior change (25, 26); and meta-analyses suggesting that narratives increase persuasiveness of health-related messaging (27, 28). Indeed, self-guided SSIs adhering to this design framework have shown consistent, sustained impacts on myriad factors that motivate adaptive behavior change, including hope (17); self-efficacy and perceived agency (19, 29); and expectancies that changes in emotions and behaviors are possible (12, 30, 31). Moreover, evidence from SSI trials suggests that short-term changes in these outcomes (e.g., perceived control and agency) predicts larger improvements in long-term clinical outcomes (e.g., depression, anxiety), suggesting these targets as likely mechanisms of SSI effects (29). Notably, all four of these design principles may be integrated into even the briefest of SSIs, including those that have required just 5–8 min of users' time (e.g., *via* inclusion of a single peer quotation, a single free-response item, or a two-sentence description of a psychoeducational concept). The SSI design features highlighted here reflect recommendations for framing SSI content, which may be built-out as briefer or longer interventions, per context-specific needs. At the same time, it is not necessarily required that an SSI encompass all four design features; they are presented as one of potentially many approaches to constructing single-session programs that spur improvements in relevant outcomes.

**Figure 1:**
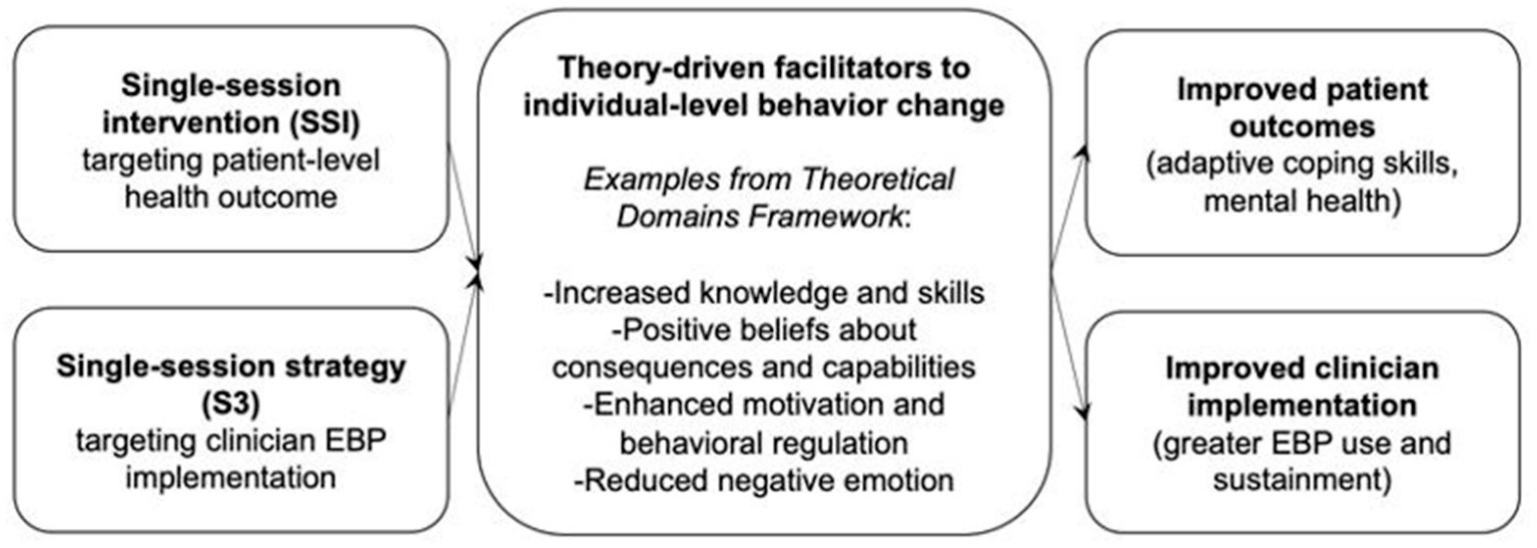
Conceptual model illustrating how Single-Session Interventions (targeting patient-level outcomes) and single-session strategies (targeting clinician EBP implementation) may shape distal outcomes of interest (patient health; clinician EBP use) by proximally shaping shared determinants to individual-level behavior change. Notably, mechanisms theorized to underlie the effects of SSI and S3 are shared, but S3 may be evaluated as a means of improving uptake of any EBP, including and beyond SSIs.

Because the mechanisms underlying SSI effects reflect generalizable drivers of behavior change, and given SSIs' consistent impacts on myriad outcomes, it stands to reason that SSIs may be helpfully reconceptualized as single-session strategies (S3) for implementation: Targeted, theory-informed activities aimed at promoting the uptake and sustainment of evidence-based clinical practices among clinicians. In other words, by tailoring the content of SSIs to address clinicians rather than patients, these brief, potent activities may be harnessed to motivate clinician EBP uptake and use (see Figure 1 for conceptual model).

Mapping overlaps between SSI capacities and widely-used implementation science frameworks may streamline tests of their utility and provide insights on development of strategies. Below, we describe how the single-session approach may be usefully integrated with the Theoretical Domains Framework (TDF), highlighting opportunities for the TDF to guide design and evaluation of single-session strategies (S3) to efficiently disseminate efforts to support clinician-level behavior change.

Notably, S3s would differ substantially in their goals and structures from existing provider-directed EBP training programs, which generally aim to establish mastery and uptake of complex, multi-pronged interventions (e.g., trauma-focused cognitive behavioral therapy) (32). Likewise, S3s would differ considerably from existing online continuing education (CE) courses for providers, which are often didactic, impersonal, lengthy (multiple hours) and often focused primarily (or exclusively) on knowledge-building (33, 34). Unlike these existing provider-directed supports, S3s would likely target uptake of highly specific EBPs (as no 15-min program can reasonably teach providers to deliver entirely new forms of treatment); and they would be designed as streamlined, interactive, and user-informed activities, in contrast to existing, highly-didactic online CE programs. Therefore, viewing S3s as activities that might be embedded within or alongside more extensive CE programs or provider trainings, along with system-level approaches to facilitating individual-level change, might be more fruitful than viewing S3s as alternatives to existing, provider-directed training programs.

## Understanding single-session strategies for implementation using the Theoretical Domains Framework

The TDF is a leading implementation determinant framework that incorporates 128 constructs spanning 12 domains, derived from 33 different theories of behavior change (11). The TDF organizes myriad constructs known to motivate individual-level behavior change. For the purposes of this Perspective—and given the known best-uses for SSIs—we focus here on individual-level factors within the TDF (35). Individual-level TDF domains include knowledge (e.g., of scientific rationale for implementation); skills (e.g., ability); social/professional role and identity (e.g., group norms); beliefs about capabilities (e.g., self-efficacy); beliefs about consequences (e.g., outcome expectancies); motivation and goals (e.g., intention); memory, attention, and decision processes (e.g., attention control); emotion (e.g., burnout); behavioral regulation (e.g., feedback); and nature of the behavior (e.g., routine). These factors may be considered as individual, mechanistic targets for implementation strategies for mitigating individual-level barriers to behavior change—any of which might be tested as possible change mechanisms in future implementation research, per recent calls by leaders in the field (7).

Based on the SSI literature, it stands to reason that S3 for implementation—built as brief, streamlined programs for clinicians to complete—may be able to target multiple TDF-identified targets for individual behavior change. Table 1 overviews how previously developed SSIs (targeting patient-level outcomes) have targeted each of the TDF's individual-level barriers.

**Table 1:** Mapping individual-level TDF-defined behavior change determinants onto single-session implementation strategy (S3) targets.

**TDF determinant**	**Targetable *via* S3?**	Examples from evidence-based Single-Session Interventions targeting individual-level outcomes^*^
Knowledge	Yes	A parent-directed SSI provides psychoeducation about child anxiety, including a scientific rationale for reducing parenting behaviors that accommodate children's avoidance of anxiety-provoking stimuli, and encouraging approach-related (“brave”) behaviors instead (15) SSI length: 30 min Format: Digital (self-guided) Primary outcome(s): Parent accommodation of child anxiety
Skills	Yes	An adolescent-directed SSI teaches, and embeds opportunities users to rehearse, “behavioral activation:” an evidence-based strategy for increasing positive affect by engaging in values-aligned activities (17, 23) SSI length: 5–20 min Format: Digital (self-guided) Primary outcome(s): Depressive symptoms; hopelessness
Social and professional role and identity	Yes	An adolescent-directed SSI includes survey results suggesting that >95% of their peers report *at least some* difficulty making friends at the start of a new school year, and that most report making *at least one close friend* by the end of that same year, normalizing and instilling hope among users (19). SSI length: 20 min Format: Digital (self-guided) Primary outcome(s): Depression, anxiety
Beliefs about capabilities	Yes	A college student-directed SSI is designed to instill the belief that empathy is a malleable skill that one can develop with practice, as opposed to a fixed trait that people “have or don't” (12). SSI length: 30–60 min Format: Digital (self-guided) Primary outcome(s): Empathy malleability beliefs; Empathic accuracy
Beliefs about consequences	Yes	An adult-directed SSI teaching that emotions are malleable through effort, as opposed to fixed and uncontrollable, increases expectancies that psychotherapy could be effective in treating mental health problems (31). SSI length: 5–8 min Format: Digital (self-guided) Primary outcome(s): Expectancies for the effectiveness of psychotherapy
Motivation and goals	Yes	An adolescent-focused SSI increased intentions to stop self-harming behaviors among youth with a recent history of non-suicidal self-injury (16, 17). SSI length: 5–30 min Format: Digital (self-guided) Primary outcome(s): Intentions to stop self-harming; Non-suicidal self-injury
Memory, attention, and decision processes	Yes	A Single-Session Intervention teaching users to practice mindful, non-judgmental awareness of chronic pain (i.e., supporting *attentional control*) significantly reduces pain catastrophizing, pain interference, and pain intensity among adults with chronic lower back pain (18). SSI length: 120 min Format: Provider-delivered Primary outcome(s): Pain catastrophizing, pain interference, pain intensity
Emotion	Yes	Multiple adolescent- and adult-directed SSIs reduce hopelessness, depression, and anxiety symptoms, both immediately and across multi-month follow-ups (15, 17, 19, 20, 23). SSI length: 5–60 min Format: Digital (self-guided) and provider-delivered Primary outcome(s): Depression, anxiety
Behavioral regulation	Yes	A parent-directed SSI provides immediate feedback (and opportunities to self-correct) during quizzes and vignette-based tasks, in which parents are asked to identify evidence-based strategies for reducing anxiety and promoting bravery in their children (15). SSI length: 30 min Format: Digital (self-guided) Primary outcome(s): Parent accommodation of child anxiety
Nature of the behavior	Yes	An adult-directed SSI supports the creation of a personalized “action plan” to support the implementation of concrete, daily steps toward a values-aligned goal. Individuals select *when, where*, and *with whom* they will implement each goal-aligned step, resulting in a documented routine for them to follow in the future (22). SSI length: 45–60 min Format: Provider-delivered Primary outcome(s): Hopelessness; Perceived agency/self-efficacy

* All examples are drawn from clinical trials or randomized experiments of SSIs, in which the SSI was found to significantly improve the primary patient-/individual-level outcome of interest (e.g., parenting behaviors; expectancies for therapy effectiveness; mental health symptom severity).

Notably, several examples in Table 1 reflect SSIs that primarily target one (or just a few) TDF-derived barriers. However, it is also possible for an SSI to simultaneously address multiple TDF-identified barriers, without substantially increasing intervention length. One example is the ABC (“Action Brings Change”) Project: a 20-to-30-min, self-guided digital SSI based on principles of behavioral activation, an evidence-based depression intervention [the ABC Project was recently redesigned as a 5-to-8 min self-guided program, without demonstrating any reductions in proximal or distal effects—suggesting that its potency and capacity to target mechanisms of change does not depend on longer program duration (17)]. ABC was designed for adolescents experiencing depression; the program encourages users to “take action” in moments of sadness and amotivation by engaging in values-aligned activities (23). It has significantly reduced depressive symptoms in high-symptom teens relative to a placebo control (19). The follows the four SSI design features noted above (knowledge provision; user empowerment; personal narratives; advice-giving opportunities), which in this case easily map onto various TDF-derived barriers. First, the program addresses knowledge *via* psychoeducation about the nature of depression, and how taking values-based actions can boost mood in moments of low motivation or distress. It simultaneously targets social identity by providing users with norms regarding the many teens who experience depression—along with symptom relief after practicing values-based actions. It addresses skills and the nature of the behavior through a personalized “action plan,” wherein users build a plan for engaging in specific, values-aligned activities in response to negative emotions. Further, it enhances memory for SSI content, empowering users to advise a peer in “taking action” to manage their mood [such “self-persuasion” writing activities promote internalization of novel beliefs (36)]. ABC has shown positive effects on beliefs about capabilities and consequences [e.g., increased confidence in one's capacity to cope with depression-related challenges (22)], and emotions [e.g., reduced hopelessness and depression symptoms (19)].

Overall, viewing the ABC Project within the TDF framework helps clarify the individual-level behavior change barriers, or mechanisms (7), through which the program might shape patient-level outcomes. By including assessments of proximal outcomes at immediate pre- and post-SSI along with distal clinical outcomes, prior SSI trials have identified the mechanisms (among those targeted) most likely to underlie effects on future symptom reductions (here, increased beliefs about capabilities and consequences and more positive emotions). Therefore, even when an SSI might be viewed as targeting multiple mechanisms simultaneously, it remains possible—through thoughtful and well-timed assessment—to parse which mechanisms matter most. The TDF also allows for parsing strengths and gaps in the broader SSI literature: Which TDF-derived barriers should an SSI target to maximize impacts on target outcomes? How do best-fit TDF targets vary across settings and behavior change goals? Future program development and evaluation may clarify these and related questions.

## Applying TDF to build, optimize, and test single-session strategies for implementation

What might a S3 for clinician EBP implementation look like, in practice, and how might the TDF inform its design? We offer an example of what an S3 might include, and how it might theoretically integrate with implementation strategies at other levels.

For illustrative purposes, a helpful context to consider is primary care: the first, and often only, healthcare access point for large portions of the population. One EBP for which primary care providers may benefit from implementation support involves providing patients with evidence-based mental health treatment recommendations, for those presenting with psychiatric difficulties. Although at least one implementation approach has been designed to support uptake of this EBP among primary care physicians (37), it is highly time- and resource-intensive—nearly 4 h long and designed for delivery by health professionals—and was not designed to target TDF-guided behavior change principles. Accordingly, we consider what a theory-driven, scalable S3 targeting this EBP might look like, if we rebuilt it based on the aforementioned SSI design principles and TDF-identified determinants.

First, the S3's delivery format is important to consider. Meta-analytic evidence suggests that effect sizes for clinician-delivered and digital (fully self-guided) SSIs for youth mental health do not significantly differ from one another, and several self-guided SSIs targeting TDF-guided behavior change factors have improved patient-level outcomes (12, 14–16, 19). Because digital, self-guided strategies are inherently easier to disseminate, technology-mediated S3s seem practical to prioritize and test. Many evidence-based, patient-directed digital SSIs require between 5 and 30 min to complete (see Table 1), suggesting an approximate target duration for novel S3s targeting clinician behavior change. Moreover, constructing S3s as self-guided digital activities would fit easily into many healthcare organizations' existing workflows for disseminating learning modules to clinicians (*via* digital platforms).

Second, we consider which TDF-guided behavior change targets to address, and how to address them within best-practice SSI design frameworks (as noted above, these design principles are not required to include in all SSIs or S3s; rather, they are applied here to exemplify one well-evidenced approach to designing SSIs that can spur individual-level behavior change). Toward the “providing scientific evidence” SSI design principle, the self-guided S3 might convey known benefits of making evidence-based mental health care recommendations. Drawing from the TDF, the S3 might target barriers linked to knowledge and professional/social identity by sharing data regarding norms among primary care physicians' mental health treatment recommendations to patients, along with the direct patient benefits that evidence-based treatment recommendations confer. Toward the “helping others/sharing back knowledge” and “users as experts” SSI design principles, the S3 might further address professional identity barriers by empowering physicians to write anonymous notes to others in their organization, sharing their personal and professional perspectives on the value of offering evidence-based treatment recommendations to patients with psychiatric needs. Third, toward the “testimonials from similar others” SSI design principle (and further addressing knowledge and professional barriers), the S3 might include testimonials from other physicians and patients, describing how making or receiving evidence-based mental health care recommendations benefited them personally. Through each of these approaches, targeting knowledge and professional identity-related barriers might enhance physicians' motivation to implement the new practice, as well as expectancies that doing so will benefit patients. Moreover, embedding an interactive “treatment recommendation plan” within the S3, wherein physicians select best-fit evidence-based treatment recommendations for common presenting mental health problems in their patient population (similar to the “action plan” embedded within existing SSIs (24), and resulting in a tangible resource for physicians to offer patients) might increase their perceived capability to implement the practice in real-time. Comparing S3 that target one or several of the above-mentioned TDF-identified behavior change targets and testing their relative effects on the behavior of interest (providing patients with evidence-based mental health treatment recommendations) might clarify which behavior-change barriers (and, in turn, which change mechanisms) are most important to target. Randomized factorial experiments might be useful methods for comparing the utility of targeting different combinations of behavior-change barriers *via* versions of the same S3.

It is also likely that S3 effects on clinician-level change will be enhanced if combined with implementation strategies at the organizational levels, given that implementation science focuses on clinician behavior within organizational constraints. Indeed, there will be many circumstances wherein organization-level strategies are essential to spurring initial EBP uptake—and in such cases, S3 approaches might enhance the capacity of those organization-level strategies to sustain clinician-level behavior change. This possibility opens a wide range of empirical questions to evaluate in diverse contexts of care. For instance, once an optimal S3 is developed for a given clinical context and EBP, one might compare the relative utility of implementing the S3 alone, vs. the S3 in combination with organizational- and system-level implementation strategies—for instance, providing primary care practices with intensive, expensive facilitation programs designed to support EBP uptake (38). Similar study designs have been used to test whether “nudges” are sufficient to change clinician EBP use, or whether more intensive, structural strategies are needed for nudges to sustain behavior change (39). Future research might compare the utility of an S3 relative to (or combined with) other implementation strategies—such as EMR-based reminders for physicians to recommend evidence-based mental health treatment options, yoked to individual patient diagnoses. Overall, the impacts of theory-driven S3 remain unknown—and hold considerable promise—across a wide variety of healthcare contexts, representing an important set of empirical questions to test in future research.

## Discussion

We have proposed a novel approach to developing individual-level theoretically informed and brief and scalable implementation strategies for individual-level behavior change: Single-session strategies (S3) for EBP implementation. By targeting the same generalizable behavior change strategies that underlie evidence-based SSI for patient-level outcomes, single-session strategies for implementation may spur clinician-level EBP use at scale. The most novel aspect of this approach is the brevity in which it can achieve behavior change. Grounding and testing novel S3 based on established implementation frameworks, such as the TDF (as outlined here), or other common frameworks with similar elements (40) may optimize their impact on individual-level barriers to clinician behavior change. An advantage of theoretically grounded approaches, like S3, is that they clarify how implementation strategies might work, aligned with recent calls from thought leaders about mechanisms as the next frontier in implementation science (7). This aligns with other approaches used to develop implementation strategies, including implementation mapping (41). Here, we use the TDF to offer a roadmap for researchers interested in applying and evaluating different S3 approaches in diverse contexts.

Several caveats warrant consideration. First, implementing EBPs can be incredibly challenging—and for certain EBP implementation efforts, S3s for individual-level behavior change barriers will not be enough. In this vein, we are not suggesting that S3 should replace other implementation strategies already active within organizations; however, they may represent a scalable means of streamlining individual-level efforts within complex implementation plans. Likewise, in settings where no implementation plans are feasible to implement, S3 might offer sufficient support for certain types of EBP uptake. Both possibilities require future study. Additionally, any S3 will be unable to alter structural barriers that often strongly shape clinicians' motivation and behavior (42). Thus, there is a need for research on contexts and structural factors that may catalyze or stymie S3 effectiveness.

In future examinations of TDF-guided S3s for clinicians, it will be critical to optimize program feasibility and acceptability. Patient-level SSIs are more feasible than longer-term psychotherapies for individuals to access and complete, but they still require some degree of effort and motivation from users. Across healthcare settings, clinicians have exceptionally limited time; therefore, new S3s must be brief and simple to ensure acceptability. Substantially-reduced versions of the same patient-directed, digital SSIs—from 25 to 5 min—produce comparable impacts on clinically-important outcomes [e.g., hopelessness, self-hate (17)]. Therefore, minimizing S3 user burden—and making S3 completion rewarding (e.g., offering compensation for S3 completion; integrating S3 completion into continuing medical education)—will be critical to sustaining programs.

Alongside prioritizing acceptability, co-design with clinicians in different settings will be critical to S3 success, as it has been for evidence-based patient-directed SSIs (23). Some patient-directed SSIs have shown equivalent effectiveness across diverse populations [e.g., LGBTQ+ youth (43)], but the same cannot be assumed for S3 for EBP implementation. Because salient behavior-change barriers are likely to differ across healthcare contexts, S3s that target the same EBP might require substantial adaptation across settings—and they might prove most acceptable and useful to providers at different points in long-term implementation processes. Thus, population-specific S3 co-design will remain key to effective design and dissemination.

Overall, single-session strategies (S3) for implementation represents a promising but yet-to-be-tested approach for streamlining and scaling individual-level behavior change efforts in healthcare settings. Future organization-partnered research may reveal the promise of this approach across diverse healthcare settings, contexts, and EBPs.

## Data availability statement

The original contributions presented in the study are included in the article/supplementary material, further inquiries can be directed to the corresponding author.

## Author contributions

JS and RB conceptualized the manuscript. JS wrote the initial draft. Both authors reviewed, edited, and approved of the final manuscript.

## Funding

JS receives research and grant support from NIH Office of the Director (DP5OD028123), NIMH (R43MH128075), NSF (2141710), HRSA (U3NHP45406-01-00), Upswing Fund for Adolescent Mental Health, the Society for Clinical Child and Adolescent Psychology, and the Klingenstein Third Generation Foundation. RB reported receiving grants from the National Institutes of Health, Patient Centered Outcomes Research Institute, the US Centers for Disease Control and Prevention, and the National Psoriasis Foundation outside of the submitted work. The preparation of this article was supported in part by the Implementation Research Institute (IRI), at the George Warren Brown School of Social Work, Washington University in St. Louis; through an award from the National Institute of Mental Health (R25MH080916). JS is an IRI Fellow and RB is IRI Faculty.

## Conflict of interest

JS serves on the Scientific Advisory Board for Walden Wise and the Clinical Advisory Board for Koko, is Co-Founder and Co-Director of Single Session Support Solutions. Inc., and receives book royalties from New Harbinger, Oxford University Press, and Little Brown Book Group. RB is principal at Implementation Science & Practice, LLC, she receives royalties from Oxford University Press, consulting fees from United Behavioral Health and OptumLabs, and serves on the advisory boards for Optum Behavioral Health, AIM Youth Mental Health Foundation, and the Klingenstein Third Generation Foundation outside of the submitted work. In addition to the above potential conflicts, the authors would like to note that JS leads a research lab whose mission involves designing, evaluating, and disseminating single-session mental health interventions, including those discussed in the present manuscript.

## Publisher's note

All claims expressed in this article are solely those of the authors and do not necessarily represent those of their affiliated organizations, or those of the publisher, the editors and the reviewers. Any product that may be evaluated in this article, or claim that may be made by its manufacturer, is not guaranteed or endorsed by the publisher.
